# A Novel Allelic Variant of *OsAGPL2* Influences Rice Eating and Cooking Quality

**DOI:** 10.3390/cells14090634

**Published:** 2025-04-25

**Authors:** Yuqing Dan, Fudeng Huang, Junfeng Xu, Yong He, Ruixiao Peng, Chunshou Li, Jiayu Song, Yuanyuan Hao, Zhihong Tian

**Affiliations:** 1 Engineering Research Center of Ecology and Agricultural Use of Wetland, Ministry of Education, College of Life Science, Yangtze University, Jingzhou 434025, China; m13147119027@163.com (Y.D.); warers@yangtzeu.edu.cn (Y.H.); yuzhongprx@163.com (R.P.); 2 Institute of Crop and Nuclear Technology Utilization, Zhejiang Academy of Agricultural Sciences, Hangzhou 310021, China; huangfd@zaas.ac.cn (F.H.); lichunshou@126.com (C.L.); 15940148227@163.com (J.S.); 3 State Key Laboratory for Quality and Safety of Agro-Products, Zhejiang Academy of Agricultural Sciences, Hangzhou 310021, China; njjfxu@163.com; 4 Key Laboratory of Traceability for Agricultural Genetically Modified Organisms, Ministry of Agriculture and Rural Affairs, Zhejiang Academy of Agricultural Sciences, Hangzhou 310021, China

**Keywords:** rice quality, *OsAGPL2*, white-core endosperm mutant, superior haplotype

## Abstract

Starch biosynthesis is crucial in determining rice quality during rice endosperm development. This study obtained a stable inheritable white-core endosperm mutant, *h5*, by treating the japonica rice variety Nipponbare with MNU (N-methyl-N-nitro-sourea). The mutated gene is an allele of *OsAGPL2*, which encodes the large subunit of ADP-glucose pyrophosphorylase (AGPase), a key and rate-limiting enzyme in the rice starch biosynthesis pathway. A G-C mutation in the third exon of *OsAGPL2* led to impaired starch synthesis, significantly reduced amylose content (AC) and gel consistency (GC), and a marked decrease in AGPase activity. The haplotype analysis revealed that an SNP in the 3′UTR and two SNPs in the 5′UTR of *OsAGPL2* were associated with significant differences in AC and GC among rice resources. These SNPs can be utilized to design molecular markers for breeding programs to improve rice quality. This study elucidates the impact of OsAGPL2 on the eating and cooking quality of rice. It identifies superior haplotypes, providing a theoretical foundation and molecular markers for accumulating minor-effect genes to enhance rice quality.

## 1. Introduction

Rice is one of the most important staple crops worldwide. Beyond enhancing yield, the cultivation of high-quality rice with superior taste, texture, and nutritional value has become a shared goal for both consumers and breeders. The evaluation of rice quality primarily encompasses milling quality, appearance quality, eating and cooking quality (ECQ), and nutritional quality. Among these, ECQ, which assesses the sensory attributes, texture, and color of cooked rice, is a primary consideration for consumers when purchasing rice. Starch, constituting approximately 85% of the rice endosperm, is the main component of rice. The physicochemical properties of starch can be used to indirectly evaluate ECQ. The key indicators include amylose content (AC), gel consistency (GC), gelatinization temperature (GT), and the Rapid Visco Analyzer (RVA) profile. Among these, GT is most reliably and simply determined by the alkali spreading value (ASV) [1].

ECQ traits are quantitative and controlled by multiple major and minor genes. AC is regulated by the major gene *Wx*, with various alleles and their combinations explaining the differences in AC among different varieties [2,3,4]. *ALK/SSIIa* is the major gene regulating GT, and it is genetically linked with *Wx* [5,6]. Different alleles of *ALK* (*ALK*^a^, *ALK*^b^, *ALK*^c^) can alter the structure of amylopectin, thereby affecting GT [7]. Major quantitative trait loci (QTLs) for GC are distributed across various chromosomes in rice [8,9,10,11]. It is currently believed that GC is primarily regulated by a C-T difference in the tenth exon of *Wx* [1,12], with additional minor effects from genes like *ALK*.

In addition to these major genes, several genes directly involved in starch biosynthesis, including but not limited to *OsAGPL3*, *OsAGPL2*, *OsPHO1*, *OsGBSS/Wx*, *OsSSI*, *OsSSIIa*, *OsSSIVb*, *OsBEI*, and *OsBEIIb*, as well as genes indirectly involved in starch synthesis and metabolic regulation, can influence ECQ [13]. For example, *OsBT1* encodes an ADP-glucose transporter and regulates starch accumulation; the mutations in *OsBT1* result in floury endosperm and reduced rice quality [14]. OsLESV interacts with OsISA1 to target starch granules, co-regulating starch biosynthesis and endosperm development [15]. The transcription factor OsbZIP58 directly binds to the promoters of multiple starch biosynthesis-related genes (*Wx*, *ALK*/*OsSSIIa*, *OsBEIIbI*, *OsSA1*, *OsAGPL3*) and regulates their expression [16]. The AP2/EREBP family transcription factor RSR1 regulates *SSI* expression; the loss of RSR1 enhances the expression of starch synthesis-related genes, increases amylose content, alters amylopectin structure, and results in round starch granules [17]. The transcriptional repressor OsSGL also affects rice taste quality by modulating starch content and gelatinization properties [18].

AGPase, catalyzing the first step of starch synthesis, converts glucose-1-phosphate (G1P) into ADP-glucose (ADPG), is activated by 3-GPA, and inhibited by Pi. In rice, OsAGPase is a heterotetramer composed of two large and two small subunits, encoded by four large subunit genes (*OsAGPL1-4*) and two small subunit genes (*OsAGPS1-2*). During endosperm filling, *OsAGPL2* and *OsAGPS2b* are predominantly expressed in the endosperm and play a crucial role in starch accumulation [19]. Nonsense and missense mutations in *OsAGPL2* and *OsAGPS2b* impair the allosteric regulation of AGPase, truncate the starch synthesis pathway, and result in shrunken and floury endosperm phenotypes [20]. Among the existing *OsAGPL2* allelic mutants, some exhibit severe phenotypes with almost complete loss of function, such as *AGPL2-3*, *w24*, *gif2*, and *M37* [21,22,23,24], while others, like *ed6*, *ed7*, and *M10*, show milder phenotypes with white-core endosperm due to single-base mutations [25,26]. These allelic mutants display varying degrees of phenotypic and physicochemical changes in starch, highlighting the significant regulatory role of *OsAGPL2* in grain development and rice quality formation.

To evaluate the potential application of *OsAGPL2* in rice quality improvement breeding, an *OsAGPL2* mutant, *h5* (in a *japonica* rice ‘Nip’ background), with white-core endosperm was obtained. The mutant exhibited a significant decrease in both AC and GC. The haplotype analysis revealed that one SNP in the 3′UTR and two SNPs in the 5′ UTR of *OsAGPL2* significantly affected AC and GC in rice resources, respectively. These superior haplotypes could be used to develop molecular markers, providing a theoretical basis for marker-assisted breeding of *OsAGPL2*.

## 2. Materials and Methods

### 2.1. Plant Materials and Growth Conditions

The Nipponbare (Nip, *Oryza sativa*, ssp. *japonica*), N22 (*Oryza sativa*, ssp. *indica*), *h5* mutant and 537 rice accessions (screening of the germplasm repository in the RFGB database (https://rfgbv2.rmbreeding.cn/, accessed on 10 March 2022) used in this study were grown in Hangzhou, Zhejiang Province, China (30.3° N, 120.2° E) and Lingshui, Hainan Province, China (18.48° N, 110.02° E). Mature seeds and other tissues were harvested according to the experimental requirements. The *h5* mutant was initially generated by treating Nip flowering spikes with 1 mM N-methyl-N-nitro-sourea (MNU) (Sigma, St. Louis, MO, USA) solution for one hour. Through two generations of self-pollination, a stably inherited M_2_ mutant line of *h5* was established.

### 2.2. Determination of Starch Physicochemical Properties

A total of 250 g of mature wild-type and *h5* seeds were dehulled, and the aleurone layer and embryo were removed. The seeds were then ground into rice flour, sieved through a 150-mesh screen, and dried for further use. Each sample was measured three times.

The total starch was measured using a starch assay kit (Megazyme, Dublin, Ireland).

AC was evaluated according to the previous report [27]. A 0.02 g sample was mixed with 0.15 mL of 95% ethanol and 1.35 mL of 1 mol/L potassium hydroxide, then incubated at 50 °C for 20 h. The mixture was diluted to 15 mL and thoroughly mixed. A 100 μL aliquot was taken, acidified with 20 μL of acetic acid, mixed with 30 μL of iodine solution, and diluted to 2 mL. After standing for 20 min, the absorbance was measured at 620 nm using a spectrophotometer UV2600 (Shimadzu, Kyoto, Japan). A standard curve was established using standard samples with known AC, and the AC of the samples was calculated based on this curve.

GC was determined according to the previous report [10]. A 100 mg sample was placed in a 13 mm × 100 mm test tube, mixed with 200 μL of 95% ethanol and 0.2 mol/L potassium hydroxide solution, and heated in a boiling water bath for 8 min. After standing at room temperature for 5 min, the tube was cooled in an ice bath for 20 min. The tube was then placed horizontally in a temperature-controlled incubator calibrated with a level. After 1 h, the distance of gel migration in the tube was immediately measured.

GT was determined using the ASV method according to the previous report [28]. Six intact grains were placed in a plastic box (5 × 5 × 2.5 cm) with 10 mL of 1.7% KOH solution, ensuring the grains were well dispersed. The box was covered and incubated in a 30 °C oven for 23 h. Based on the ASV score, the rice grains were classified into four groups: high (1–2), medium-high (3), medium (4–5), and low (6–7).

The pasting properties of rice flour were determined using a Rapid Visco Analyzer (RVA, Perten Instruments, Sydney, Australia) following the standard method described by [29]. A 3.0 g sample of rice flour was weighed into a specialized aluminum canister, mixed with 25 g of distilled water, and placed in the RVA instrument, which had been preheated for at least half an hour. The test program was as follows: hold at 50 °C for 1 min; linearly increase the temperature to 93 °C over 5.5 min; hold at 93 °C until 7 min; linearly decrease the temperature to 50 °C by 11 min; and hold at 50 °C for the remaining 12.5 min. RVA data were recorded.

Urea swelling was evaluated according to the previous report [29]. A 20 mg sample was mixed with 1 mL of 0–9 M urea solution. The pH of the solution was adjusted to 6.0 using acetic acid in a centrifuge tube. The mixture was incubated at 25 °C for 24 h, then centrifuged at 8000× *g* for 20 min at room temperature and allowed to stand for 1 h. The solubility of starch granules in the urea solution was determined by measuring the volume of the swollen precipitate.

### 2.3. Microscopic Analysis

The dehulled brown rice kernels and cross-sections of brown rice kernels from the mid-region were photographed using a camera (VHX 950F, KEYENCE, Tokyo, Japan).

The scanning electron microscopy (SEM) was performed on intact dry seeds of the wild-type and *h5* mutant. The samples were cross-sectioned with a blade and imaged using a Hitachi TM 3000 (Tokyo, Japan) scanning electron microscope.

The transmission electron microscopy (TEM) was conducted on endosperm tissues collected from wild-type and *h5* plants at 7 days after flowering (DAF). The samples were cross-sectioned to ~1 mm thickness and fixed in 2.5% glutaraldehyde for 12 h at 4 °C. Following ethanol gradient dehydration, samples were embedded in LR White resin (London Resin, Berkshire, UK), ultrathin-sectioned using a Leica CM1950 microtome, and examined with a Hitachi H7650 TEM (Tokyo, Japan).

The sample preparation of semi-thin sections for microscopy was similar to that for TEM. The sections (1 µm) were stained with I_2_-KI for 5 s and then observed and photographed under an optical microscope (Axio Vert.A1, Zeiss, Oberkochen, Germany).

### 2.4. Mapping of OsAGPL2

Nine individuals with extreme floury endosperm phenotypes from F_2_ generation, derived from a cross between the homozygous *h5* mutant and N22, were used for initial mapping through the 1K chip platform (Higentec, Changsha China). Simultaneously, whole-genome resequencing (Higentec, Hunan, China) of the wild-type and *h5* genomes was performed using the Nip genome as a reference to identify the target gene with amino acid changes in the initial mapping interval.

### 2.5. Haplotype Analysis

The haplotype analysis was conducted using data from 537 germplasm resources for GC and AC, combined with whole-genome sequencing results from the RFGB website. Multiple comparisons were performed using *t*-tests.

### 2.6. AGPase Enzyme Activity Assay and Sugar Component Determination

AGPase activity was detected using an AGP assay kit (Nanjing Mofan Biotechnology Co., Ltd., Nanjing, China). The content of plant sugar components was determined using the anthrone-H_2_SO_4_ method as described by [30].

### 2.7. RNA Extraction and Gene Expression Analysis

RNA was extracted 7 days after grain filling endosperms of wild-type and *h5* seeds using the MiniBEST Plant RNA Extraction Kit (TaKaRa, Tokyo, Japan). The first strand of cDNA was synthesized using a reverse transcription kit (Novoprotein, Suzhou, China). qRT-PCR was performed on a Bio-Rad real-time PCR device using a SuperMix kit (Novoprotein, Suzhou, China). The primers are listed in Table S1.

## 3. Results

### 3.1. Phenotypic Differences of WT and h5

**Figure 1:**
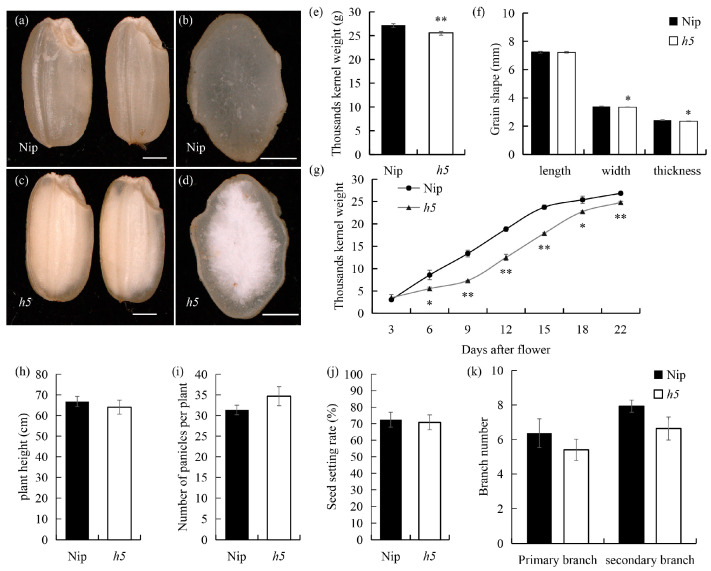
The appearance of wild-type and *h5* seeds. (**a**,**c**) A comparison of wild-type and *h5* seeds. Scale bars: 1 mm. (**b**,**d**) Cross-sections of wild-type and *h5* seeds. Scale bars: 500 µm. (**e**) The 1000-grain weight of wild-type and *h5*. (**f**) Grain length, grain width, and grain thickness of wild-type and *h5* seeds. (**g**) Grain filling of wild-type and *h5* at various developmental stages. Grain weight is the dry weight of 1000 brown rice grains. (**h**–**k**) Yield-related traits of wild-type and *h5*. Asterisks indicate statistical significance between the wild-type and *h5*, as determined by a student’s *t*-test (* *p*  <  0.05; ** *p*  <  0.01).

To identify genes affecting ECQ, the japonica rice variety Nipponbare (Nip) was mutagenized using MNU, resulting in the stably inherited mutant *h5*. Compared to the completely transparent endosperm of the wild type, the *h5* endosperm exhibited a white-core phenotype with a translucent periphery (Figure 1a–d). During endosperm development, the grain-filling rate of *h5* was significantly lower than that of the wild type (Figure 1g), leading to a significant reduction in the 1000 g weight at maturity (Figure 1e). While the grain length of *h5* remained unchanged, the grain thickness and width were significantly reduced compared to the wild type (Figure 1f). Other yield-related traits of *h5* showed no significant differences from the wild type (Figure 1h–k). These results indicate that the mutated gene affects starch synthesis in rice endosperm, thereby influencing rice quality, but has minimal impact on yield-related traits except for the 1000 g weight.

### 3.2. Significant Reduction in AC and GC in h5 Endosperm

**Figure 2:**
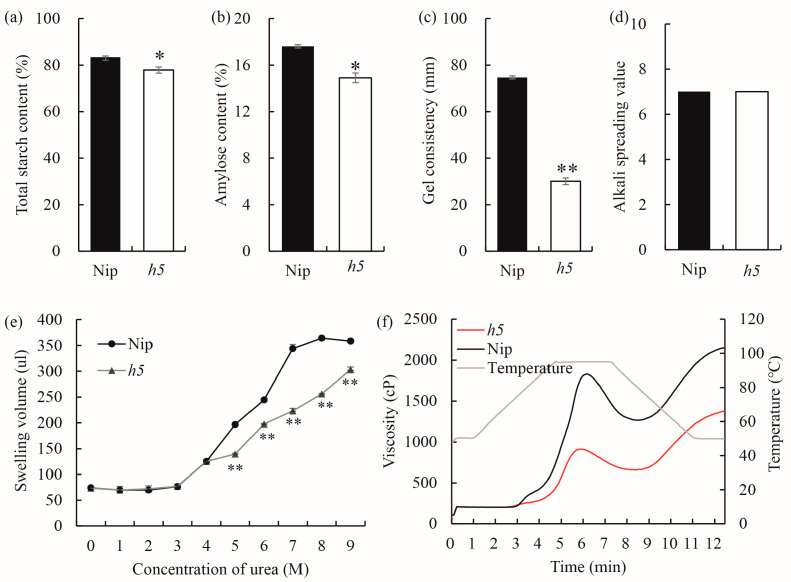
Properties and physicochemical characteristics of starch in WT and *h5*. (**a**–**d**) ECQs and the total starch content of WT and *h5*. (**e**) Volume of WT and *h5* endosperm starch swelling in different concentrations of urea. (**f**) Pasting properties of endosperm starch of WT and *h5*. Values are means ± SDs (n = 3). The asterisks indicate statistical significance between the wild type and the mutant, as determined by Student’s *t*-test (* *p* < 0.05, ** *p* < 0.01).

To investigate whether the mutated gene also affects ECQ, a series of ECQ-related parameters were measured. The results showed that, compared to the wild type, *h5* exhibited a significant decrease in total starch content and AC, as well as a highly significant reduction in GC, while the ASV showed no significant difference (Figure 2a–d). When powdered starch from *h5* and the wild type was mixed with urea solutions of varying concentrations (0–9 M), the *h5* starch exhibited less swelling than the wild type in 5 M urea and failed to gelatinize even in 9 M urea (Figure 2e). The viscosity of *h5* starch paste remained consistently lower than that of the wild type throughout the gelatinization process (Figure 2f). These results demonstrate that the mutated gene significantly alters rice ECQ.

### 3.3. Dispersed Spherical Starch Granules in h5 Endosperm

**Figure 3:**
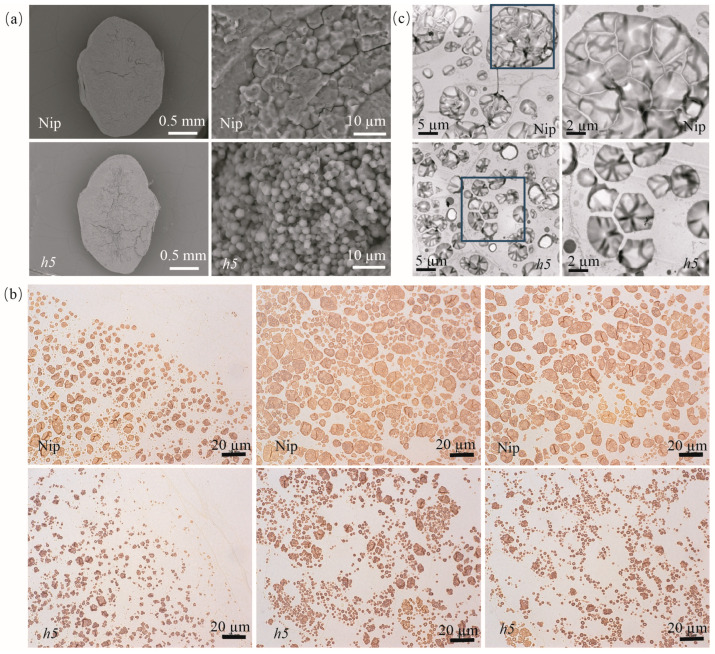
Microscopic analysis of endosperm in WT and *h5*. (**a**) SEM analysis of wild-type WT and *h5* endosperm. Scale bars: 0.5 mm for the left images, 10 µm for the right images. (**b**) Semi-thin sections of WT and *h5* endosperm at 7 DAF. The left images show the periphery of endosperm cells, while the middle and right images show the central endosperm. Scale bars: 20 µm. (**c**) TEM analysis of starch granules in wild-type and *h5* endosperm at 7 DAF. The right images are magnified views of the corresponding areas in the left images. Scale bars: 5 µm for the left images, 2 µm for the right images.

The SEM analysis revealed that the *h5* endosperm was filled with loosely packed, small, and round single starch granules, whereas the wild-type endosperm consisted of densely packed, polyhedral starch granules (Figure 3a). The TEM analysis of endosperm at 7 days after flowering (DAF) confirmed that the compound starch granules in *h5* were replaced by dispersed single starch granules (Figure 3c). The semi-thin sections further revealed an increase in single starch granules, a decrease in compound starch granules, and larger gaps between starch granules in *h5* endosperm cells, indicating delayed grain filling (Figure 3b). The transformation of compound starch granules into single starch granules and the increased gaps between them may explain the floury endosperm phenotype.

### 3.4. The Mutated Gene Is OsAGPL2

**Figure 4:**
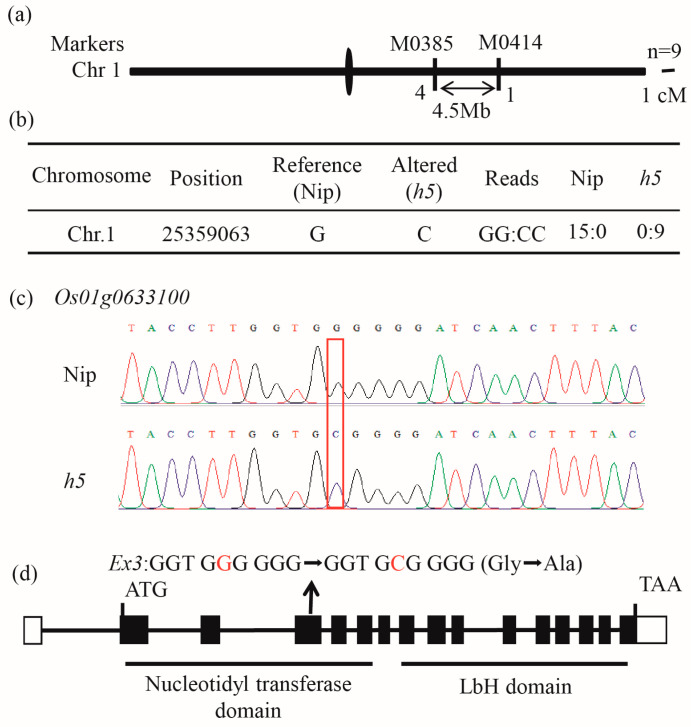
Mapping of the target gene. (**a**) Preliminary mapping of the gene using the 1K chip platform (Higentec, Hunan, China). (**b**) Resequencing (Higentec, Hunan, China) result of the wild-type and *h5* using the Nip genome as a reference to identify the amino acid changes in the initial mapping interval. The term “reads” refers to the average number of times each base is covered by reads. Nip: GG bases were covered 15 times, CC bases were covered 0 times; *h5:* GG bases were covered 0 times, CC bases were covered 9 times. (**c**) Sequencing validation of the mutation. (**d**) Gene structure and mutation site analysis. The red ‘G’ and ‘C’ mark the mutation sites.

In an F_2_ population derived from a cross between *h5* and the indica rice variety N22, nine extreme individuals with floury endosperm were selected for preliminary mapping, localizing the target gene to a 4.5 Mb interval on chromosome 1 (Figure 4a). The whole-genome resequencing of the wild type and *h5*, using the Nipponbare genome as a reference, identified a single SNP change in the gene *Os01g0633100* on chromosome 1. The wild type showed a GG:CC read ratio of 15:0 at this locus, while *h5* showed a ratio of 0:9 (Figure 4b). The sequencing confirmed the reliability of this mutation (Figure 4c). The mutation resulted in a G-to-C change in the third exon of *Os01g0633100*, leading to a glycine-to-alanine substitution at the 157th amino acid position (Figure 4d). *Os01g0633100* encodes the large subunit of AGPase, *OsAGPL2*.

### 3.5. AGPase Activity Assay, Sugar Contents Analysis and Expression Analysis of Related Genes

**Figure 5:**
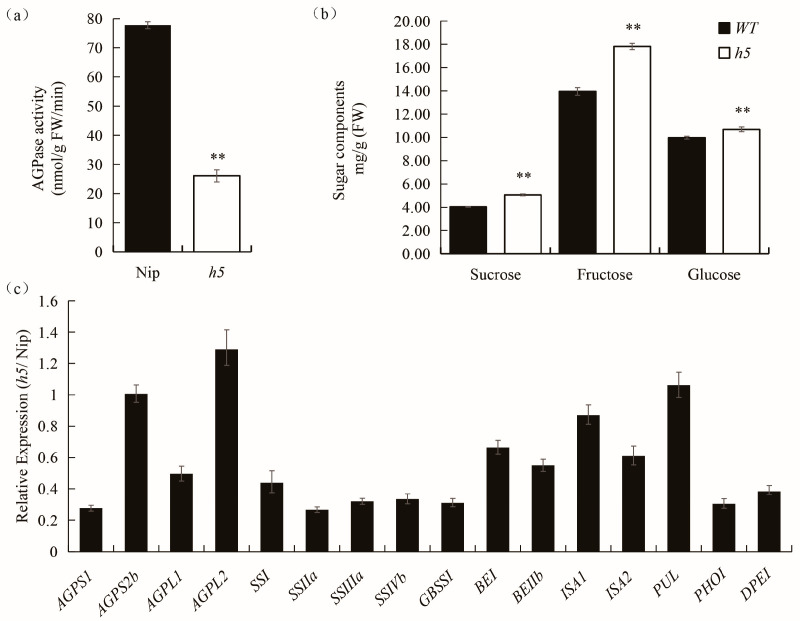
AGPase activity assay, sugar contents analysis and expression analysis of related genes. (**a**) AGPase activity assay of WT and *h5* endosperm at ~7 DAF. (**b**) Soluble sugar contents in WT and *h5* endosperm at ~7 DAF. (**c**) Expression levels of starch synthesis-related genes in WT and *h5*. Asterisks indicate statistical significance between the wild-type and *h5*, as determined by a student’s *t*-test ** *p*  <  0.01).

To investigate the effect of the *AGPL2* mutation on AGPase activity, AGPase activity was measured. The results showed a highly significant reduction in AGPase activity in *h5* (Figure 5a). Additionally, *h5* grains contained significantly increased levels of soluble sugars, including sucrose, glucose, and fructose (Figure 5b). Further analysis of the expression levels of starch synthesis-related genes revealed that the expression of *AGPL2* and *AGPS2b*, which are predominantly expressed during endosperm development, was upregulated. In contrast, the expression of other starch synthesis-related genes, except for *PUL*, was significantly downregulated (Figure 5c). These results indicate that the glycine-to-alanine mutation at position 157 of *AGPL2* indeed alters AGPase activity and disrupts starch synthesis, affecting the expression of upstream and downstream genes and resulting in pleiotropic defects in storage substance accumulation in rice endosperm.

### 3.6. OsAGPL2 Is Associated with AC and GC

The ECQ of *h5* was significantly altered compared to the wild type. To identify superior haplotypes of *AGPL2* controlling rice ECQ, the ECQ of 537 rice accessions from the RFGB resource library was measured, and haplotype analysis was performed. The results showed that only one SNP was present in the coding sequence (CDS) of *AGPL2* among the accessions, and it did not alter the amino acid sequence, indicating high conservation of the coding region.

**Figure 6:**
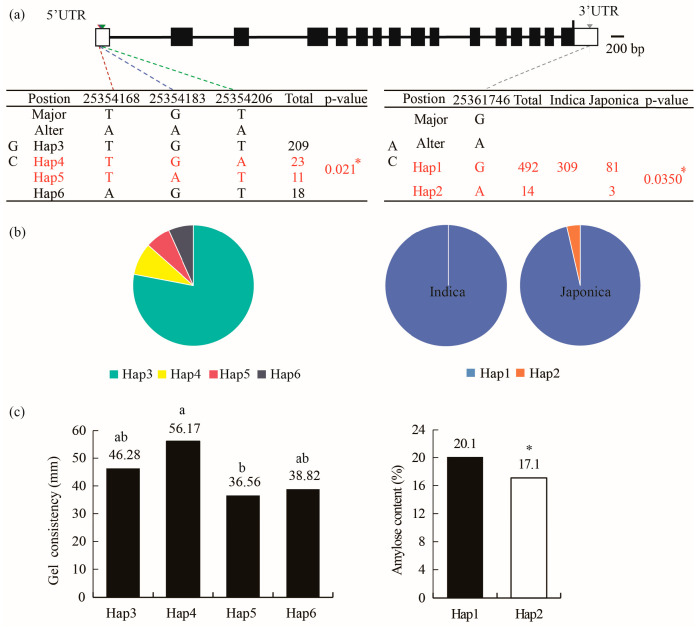
Haplotype analysis using sequencing data from RFGB and ECQ data from 537 rice accessions. (**a**) Analysis of GC and AC differences in the indica population and all resources, respectively. Three SNPs in the 5′UTR divided most indica resources into four haplotypes (Hap3-6). Accessions carrying Hap4 and Hap5 showed highly significant differences in GC. One SNP in the 3′UTR divided most accessions into two haplotypes (Hap1-2). Accessions carrying Hap1 and Hap2 showed highly significant differences in AC. * indicate significant differences by Tukey’s *t*-tests (*p* < 0.05). (**b**) Distribution of Hap3-6 in indica accessions and Hap1-2 in all accessions. (**c**) Comparison of significant differences in GC among accessions carrying Hap3-6 and significant differences in AC among accessions carrying Hap1-2. a,b and * indicate significant differences by Tukey’s *t*-tests (*p* < 0.05).

Further analysis of the 3′UTR of *OsAGPL2* revealed a G-to-A SNP at position 25361746. Accessions carrying the G allele were designated Hap1, and those carrying the A allele were designated Hap2 (Figure 6a). Hap1 was present in 97% of the accessions (Figure 6b), and accessions carrying Hap2 exhibited significantly lower AC than those carrying Hap1 (Figure 6a,c). This suggests that the 3% of accessions carrying Hap2 represents superior germplasm for reducing AC in rice quality improvement.

The analysis of the 5′UTR of *OsAGPL2* identified three SNPs at positions 25354168 (T-to-A), 25354183 (G-to-A), and 25354206 (T-to-A). Based on these SNPs, four major haplotypes were identified among the accessions (Figure 6a), with Hap3 being the most prevalent (Figure 6b). Further analysis using the indica subpopulation revealed that accessions carrying Hap4 exhibited significantly longer GC than those carrying Hap5 (Figure 6c). Notably, only when both SNPs at positions 25354183 and 25354206 were altered did GC change significantly, while the SNP at position 25354168 had no significant effect on GC (Figure 6a,c). This suggests that accessions carrying Hap4 represent superior germplasm for improving GC in rice quality enhancement.

## 4. Discussion

### 4.1. Impaired Starch Biosynthesis Pathway in the h5 Endosperm

The grain-filling stage is a critical period for the formation of rice yield and quality. Floury endosperm mutants are valuable resources for studying the starch biosynthesis pathway. In recent years, a series of rice mutants with varying degrees of floury or chalky endosperm have been reported, and their gene functions have been extensively studied [31,32]. The *h5* mutant, a white-core endosperm mutant (Figure 1) induced by MNU in japonica rice Nip, carries an allelic mutation in *OsAGPL2* (Figure 4). Its compound starch granules are disrupted, dispersing into numerous single starch granules with a rounded morphology (Figure 3). The grain-filling rate is significantly reduced, and starch accumulation and synthesis are abnormal, (Figure 1 and Figure 3) consistent with previously reported phenotypes of *OsAGPL2* mutations that alter starch granule morphology [21,23,24,25,26,33]. Among the known *OsAGPL2* mutants, the degree of flouriness or chalkiness varies depending on the rice genetic background and the specific mutation site. For example, the loss-of-function mutant *gif2*, with a 2-bp deletion causing premature termination, exhibits severe shriveled and floury endosperm [23]. Single-base mutations can produce floury and shriveled mutants, such as *w24* and *M37*, as well as weak mutants with white-core and slightly shriveled endosperm, such as *ed6*, *ed7*, *AGPL2-3*, and *M10* [21,23,24,25,26,33]. The *h5* mutant in this study displays a large white-core endosperm with a translucent periphery and slight shriveling, representing a weak mutant. The mutation site involves a glycine-to-alanine substitution, resulting in a new allelic variant of *AGPL2*. These findings suggest that mutations at different positions in the *AGPL2* coding region can cause varying degrees of endosperm developmental disorders.

*OsAGPL2* contains two domains: the nucleotide transferase domain (NTP) and the left-handed parallel beta helix (LbH) domain. Sequencing results indicate that the *h5* mutant has a single-base (G-to-C) substitution in the third exon of *OsAGPL2* (Figure 4). This site is located in the NTP domain, where conformational changes can significantly affect AGPase activity [22]. Allelic mutants such as *shr1a*, *w24*, and *M37* also exhibit amino acid changes in this domain, with the *h5* mutation site being only one amino acid away from that of *w24* [22,24,25]. Similar to these mutants, *h5* shows a significant reduction in AGPase activity and altered expression levels of starch synthesis-related genes (Figure 5), underscoring the critical role of this domain in AGPase function.

### 4.2. Three SNPs in the 3′UTR and 5′UTR of OsAGPL2 as Key Superior Loci for Rice Quality Improvement

The diversity of haplotypes in the rice genome is a valuable resource for breeding improvement, and the utilization of superior haplotypes can accelerate the process of rice quality enhancement. In the *h5* mutant endosperm, both AC and GC are significantly reduced (Figure 2), similar to the mutants *OsAGPL2-3* and *gif2* [21,23], indicating that *AGPL2* is a key gene regulating AC and GC. To further analyze whether superior haplotypes of this gene exist in rice germplasm for ECQ regulation, a haplotype analysis was conducted using ECQ data from 537 rice accessions in the RFGB database. Based on the classification of germplasm resources from RFGB, 537 rice accessions were classified into japonica, indica, and other germplasms. The result shows that the coding region of *OsAGPL2* is highly conserved, with only one SNP that does not alter the amino acid sequence.

The 3′UTR is a critical region for mature transcript formation. A G-to-A SNP at position 25361746 in the 3′UTR is associated with AC variation. Accessions carrying Hap2 exhibit significantly lower AC than those carrying Hap1. According to the RFGB classification, 97% of the accessions in this study carry Hap1, while Hap2 is predominantly found in non-indica accessions, and very few in japonica, indicating an uneven distribution of this SNP among rice germplasm (Figure 6a). Indica varieties generally have higher AC than japonica and other types [34,35]. In addition to the major effect of the *Wx* gene on AC differences between indica and japonica, the Hap1 in the 3′UTR of *AGPL2* may also contribute to the high AC in indica as a minor-effect locus. Therefore, accessions carrying Hap2 represent superior germplasm for reducing AC in rice quality improvement.

The 5′UTR influences translation initiation rates. Analysis of the 5′UTR of *OsAGPL2* identified three SNPs at positions 25354168 (T-to-A), 25354183 (G-to-A), and 25354206 (T-to-A). Based on these SNPs, the accessions were divided into four major haplotypes, Hap3-6 (Figure 6a). The haplotype analysis revealed no significant differences in ECQ traits among haplotypes when analyzing all accessions or japonica accessions. However, when analyzing indica accessions, Hap4 and Hap5 showed highly significant differences in GC. The major locus controlling GC is a C-to-T SNP in the tenth exon of *Wx* [12]. Japonica and other types almost exclusively carry the C allele associated with long GC, while indica accessions are evenly split between the C and T alleles, reflecting evolutionary divergence in indica and conservation in other types. Similarly, Hap4 and Hap5 of *AGPL2* only show significant GC differences in indica, suggesting that indica rice is more amenable to molecular marker-assisted breeding for GC improvement. Furthermore, only when both SNPs at positions 25354183 and 25354206 are altered do significant changes in GC occur, highlighting the synergistic effect of these two loci in GC improvement. Therefore, accessions carrying Hap4 represent superior germplasm for enhancing GC in rice quality improvement.

## 5. Conclusions

This study focuses on a systematic investigation of OsAGPL2, the major subunit of AGPase—a key rate-limiting enzyme in the starch biosynthesis pathway. As a critical regulator of starch accumulation, OsAGPL2 influences ECQ traits, yet remains underutilized in rice quality breeding programs. Through the obtainment of an *OsAGPL2* mutant *h5*, we comprehensively analyzed its effects on plant and grain morphology, starch granule structure, ECQ indexes, AGPase activity, sugar contents, and expression of starch biosynthesis-related genes. Furthermore, by examining 537 rice germplasm accessions from the RFGB database, we identified one SNP in the 3′UTR and two SNPs in the 5′UTR region associated with AC and GC, respectively. These findings provide valuable molecular markers for rice quality improvement. This study reveals the multifaceted regulatory roles of OsAGPL2 in rice quality formation, offering novel theoretical insights and genetic resources for both starch biosynthesis research and rice quality improvement breeding.

## Data Availability

Data is contained within the article or Supplementary Material.

## Supplementary Materials

The following supporting information can be downloaded at: https://www.mdpi.com/article/10.3390/cells14090634/s1, Table S1: Primers used in this study.
