# Supplementary material for: A Novel Allelic Variant of OsAGPL2 Influences Rice Eating and Cooking Quality
Source: Cells. 2025 Apr 25;14(9):634. doi: 10.3390/cells14090634 (PMC12071516; doi:10.3390/cells14090634)
Supplement: Supplementary file 1 [file cells-14-00634-s001.zip › supplementary.pdf]

Table S1: Primers used in this study

Name	Sequence(5'-3)
Actin1-F	TGCTATGTACGTCGCCATCCAG
Actin1-R	AATGAGTAACCACGCTCCGTCA
AGPS1-F	GTGCCACTTAAAGGCACCATT
AGPS1-R	CCCACATTTCAGACACGGTTT
AGPS2b-F	AACAATCGAAGCGCGAGAAA
AGPS2b-R	GCCTGTAGTTGGCACCCAGA
AGPL1-F	GGAAGACGGATGATCGAGAAAG
AGPL1-R	CACATGAGATGCACCAACGA
AGPL2-F	AGAGGTGCTITTGGTGATGGA
AGPL2-R	TAGCCCTTGTCCTTGTCAGA
SSI-F	GGGCCTTCATGGATCAACC
SSI-R	CCGCTTCAAGCATCCTCATC
SSlla-F	GCTTCCGGTTTGTGTGTTCA
SSlla-R	CTTAATACTCCCTCAACTCCACCAT
SSIlla-F	GCCTGCCCTGGACTACATTG
SSlla-R	GCAAACATATGTACACGGTTCTGG
SSIVb-F	ATGCAGGAAGCCGAGATGTT
SSIVb-R	ACGACAATGGGTGCCAAGAT
GBSSI-F	AACGTGGCTGCTCCTTGAA
GBSSI-R	TTGGCAATAAGCCACACACA
BEI-F	TGGCCATGGAAGAGTTGGC
BEI-R	CAGAAGCAACTGCTCCACC
BEllb-F	ATGCTAGAGTTTGACCGC
BEllb-R	AGTGTGATGGATCCTGCC
ISA1-F	GGGTCAATTTCGCCGTCTAC
ISA1-R	TACAGCATGTTGTGCAGCTC
ISA2-F	TAGAGGTCCTCTTGGAGG
ISA2-R	AATCAGCTTCTGAGTCACCG
PUL-F	ACCTTTCTTCCATGCTGG
PUL-R	CAAAGGTCTGAAAGATGGG
Pho1-F	TTGGCAGGAAGGTTTCGCT
Pho1-R	CGAAGCCTGAAGTGAACTTGCT
DPE1-F	TCTGTCGCTTGAACTTACAGAA
DPE1-R	TTTGGCTGCAAGTATTGTTCT
